# Comparison of Load Distribution in Subperiosteal Implants and Mini Plates in Orthognathic Surgery

**DOI:** 10.7150/ijms.111111

**Published:** 2025-04-22

**Authors:** Metin Berk Kasapoglu, Zeynep Afra Akbiyik Az

**Affiliations:** Istanbul University, Faculty of Dentistry, Department of Oral and Maxillofacial Surgery, Istanbul, Türkiye.

**Keywords:** orthognathic surgery, subperiosteal implants, mini plates, finite element analysis, maxillary atrophy

## Abstract

**Background:** Orthognathic surgery is important for correcting craniofacial deformities and restoring occlusion. However, in edentulous patients with severe maxillary atrophy, traditional fixation methods, such as mini plates, may not provide sufficient stability and support for prosthetic rehabilitation. Advances in additive manufacturing have enabled the development of patient-specific subperiosteal implants, offering improved biomechanical performance and more favourable load distribution.

**Methods:** This study utilized finite element analysis to compare the biomechanical performance of traditional mini plates and customized subperiosteal implants in maxillary orthognathic surgery. Computed tomography data were used to construct patient-specific models, and a Le Fort I osteotomy with a 9-mm maxillary advancement was simulated. Displacement and stress distribution were analysed under vertical and oblique loading conditions, focusing on critical regions such as osteotomy sites and screw interfaces.

**Results:** Subperiosteal implants exhibited superior biomechanical performance, with significantly lower displacement (0.58 mm) compared to mini plates (4.50 mm). Stress levels in mini plates frequently exceeded the yield strength of grade IV titanium, whereas subperiosteal implants remained within the elastic limit of Ti6Al4V. Additionally, screw stresses were reduced by 38-42% in the subperiosteal implant group, thereby reducing the risk of mechanical failure.

**Conclusions:** Customized subperiosteal implants provided enhanced stability, reduced stress concentrations, and improved load distribution compared to traditional mini plates. These findings highlight their potential as a transformative solution in orthognathic surgery, particularly for edentulous patients with severe maxillary atrophy. Future research should focus on long-term clinical outcomes and cost-effectiveness to further establish their role in maxillofacial reconstruction.

## Introduction

Orthognathic surgery is the cornerstone of the treatment of congenital and acquired craniofacial deformities, encompassing procedures such as Le Fort I osteotomy and sagittal split ramus osteotomy to correct jaw discrepancies, realign the maxilla and mandible, and restore occlusion 1,2. Traditionally, postoperative stabilization of jaw segments has relied on fixation devices such as screws and plates composed of grade IV titanium 3. However, in edentulous patients undergoing orthognathic surgery, postoperative dental rehabilitation presents significant challenges, often necessitating bone augmentation procedures to establish adequate support for prosthetic solutions 4,5. Moreover, severe alveolar bone resorption following tooth extraction, particularly in cases classified as class V and VI according to the Cawood and Howell classification, frequently renders standard dental implant solutions inadequate 1,5,6. Consequently, alternative techniques, including bone grafting, zygomatic implants, and pterygoid implants, are often required. While these methods offer viable solutions, they are associated with considerable complication risks, highlighting the need for innovative approaches 3,7-9.

Advancements in three-dimensional (3D) imaging and computer-aided design/computer-aided manufacturing (CAD/CAM) processes have facilitated the development of patient-specific subperiosteal implants. These implants, particularly beneficial in cases of severe bone loss, have gained prominence due to their ability to conform precisely to individual anatomical structures 4,10,11. In orthognathic surgery, the integration of custom-designed plates enabled by CAD/CAM technology has not only reduced surgical duration, but has also enhanced procedural accuracy and significantly minimized complication risks 12,13.

Maxillofacial deformities requiring orthognathic surgery are relatively common, particularly among young adults aged 18-30 years, and may result from congenital conditions, developmental disturbances, or trauma 14. Skeletal class III malocclusion, for instance, affects up to 5% of the population and often necessitates surgical intervention 15. In contrast, edentulous or severely atrophic patients, typically over the age of 50 years, experience progressive bone loss following tooth extraction, leading to complex anatomical challenges for both orthognathic surgery and prosthetic rehabilitation 16. The simulated case in this study involved a 9-mm maxillary advancement, a relatively large movement representing a small but clinically significant subset of orthognathic patients 17. This degree of advancement imposes substantial mechanical demands on conventional fixation systems, which have been shown to perform adequately in cases involving smaller movements 18.

Historically, such deformities were managed using removable dentures and bone grafting techniques, which provided limited stability and often necessitated multiple surgical interventions 19. However, advancements in imaging, design, and manufacturing have facilitated the use of custom subperiosteal implants, which serve both as fixation devices and as prosthetic support, offering an innovative solution for cases involving severe maxillary atrophy 3,20.

A novel advancement in this field is the simultaneous application of patient-specific subperiosteal implants with abutments, allowing for concurrent orthognathic surgery and dental prosthetic rehabilitation 4,21-24. This dual-purpose approach streamlines postoperative recovery and obviates the need for augmentation surgeries in atrophic jaws, enabling a direct transition to prosthetic loading. In edentulous patients undergoing orthognathic surgery, augmentation techniques have traditionally been required to achieve sufficient bone volume for prosthetic rehabilitation 21,25,26. However, this innovative dual-purpose technique eliminates the necessity for additional procedures, representing a significant breakthrough. By facilitating simultaneous maxillary advancement and prosthetic preparation, this approach enhances patient outcomes by expediting recovery and reducing the number of required surgical interventions.

This study evaluated the biomechanical performance of customized subperiosteal implants as fixation devices in maxillary orthognathic surgery, specifically in cases of severe maxillary atrophy. Using finite element analysis (FEA), the study compared these implants to traditional mini plates under various loading conditions, with a particular focus on stress distribution and displacement patterns. The findings aimed to demonstrate the potential of subperiosteal implants in advancing orthognathic surgery and dental implantology.

## Materials and Methods

This study was conducted in collaboration with Istanbul University Faculty of Dentistry and Tinus Technologies. Ethical approval was obtained from the Istanbul University Ethics Committee on 28 March 2024 (protocol number: 2024/26).

FEA was used to compare the biomechanical performance of traditional mini plates and customized subperiosteal implants in maxillary orthognathic surgery. All computational and modelling processes were executed on HP workstations equipped with Intel Xeon E-2286 processors operating at 2.40 GHz with 64 GB of ECC RAM. Maxillary bone models were developed using computed tomography (CT) data from an adult patient.

**Figure 1:**
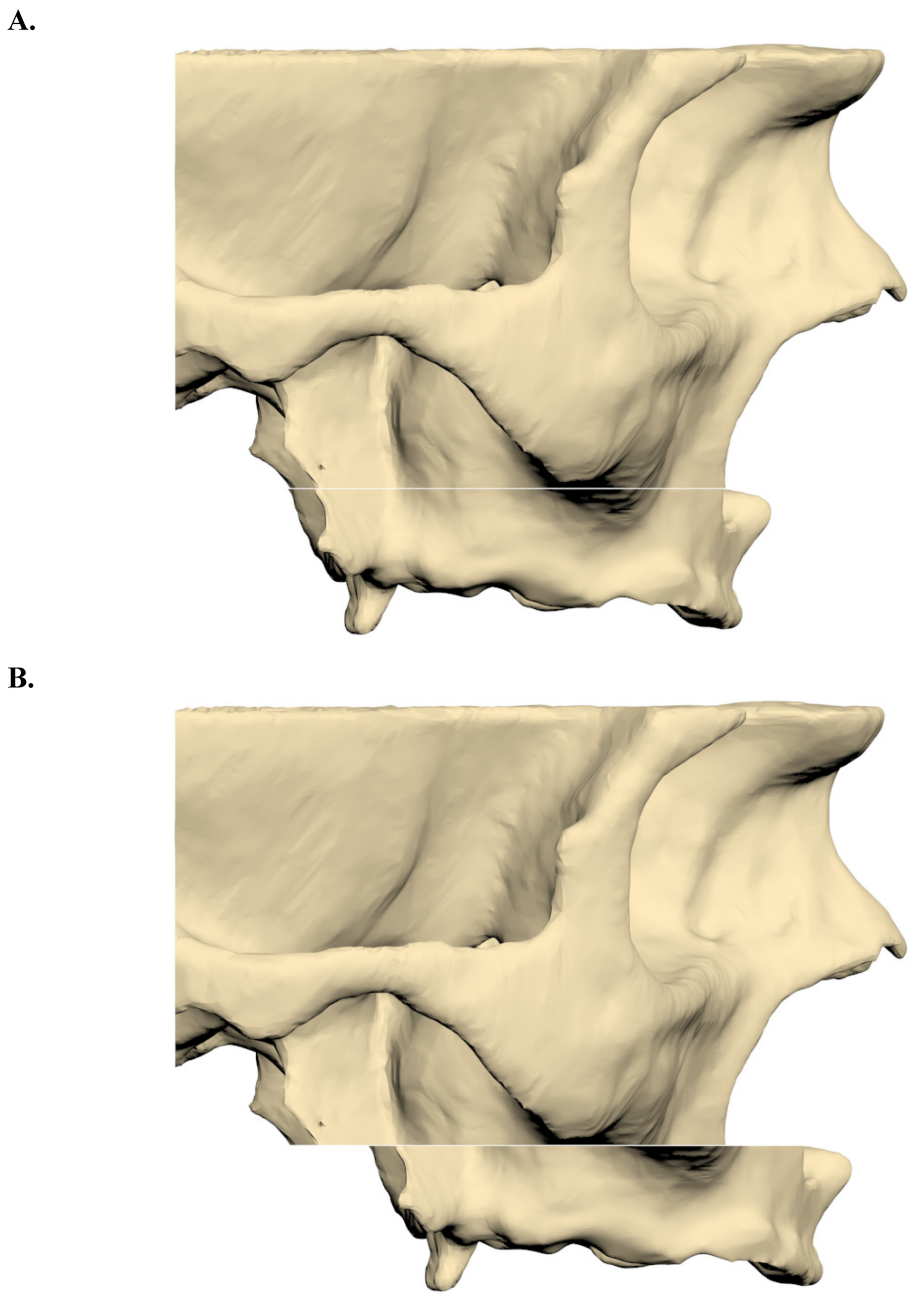
** A.** A three-dimensional maxillary bone model with a LeFort incision. **B.** After 9 mm forward advancement three-dimensional maxillary bone model.

CT images were reconstructed at a slice thickness of 0.3 mm and imported into 3DSlicer software in Digital Imaging and Communications in Medicine (DICOM) format. Image segmentation was performed using appropriate Hounsfield unit thresholds to generate 3D models, which were subsequently exported in STL format. The segmented maxillary model was then imported into ANSYS SpaceClaim software, where a Le Fort I osteotomy with a 9-mm forward advancement was simulated (Figure 1).

### Geometric model construction

**Figure 2:**
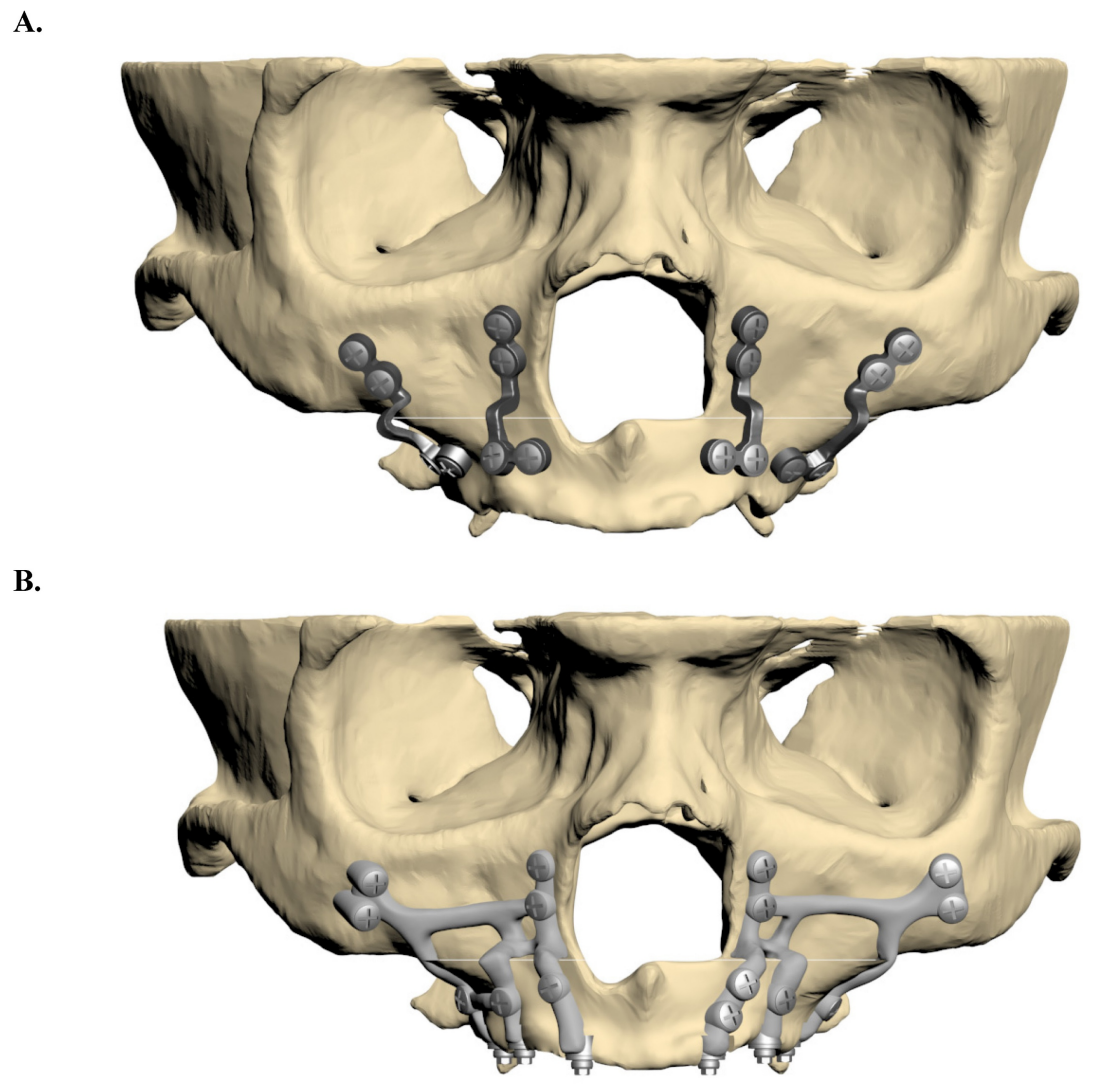
** A.** Fixation model with mini plate. **B.** Fixation model with subperiosteal implant.

Two fixation models were developed: one utilizing traditional mini plates and the other employing customized subperiosteal implants. The plates and screws were modelled according to specifications from the KLS Martin catalogue. The screws had a diameter of 2 mm and a length of 8 mm, while both the mini plates and subperiosteal implants were designed with a thickness of 2 mm. The finalized implant models were constructed in ANSYS SpaceClaim and prepared for analysis in ANSYS Workbench (Figure 2).

Geometric models were converted into finite element meshes using ANSYS Workbench, with optimization performed to ensure numerical accuracy, including convergence testing. The subperiosteal implant model consisted of approximately 2,064,403 nodes and 10,312,671 elements, whereas the mini plate model contained 1,023,285 nodes and 4,119,961 elements. Material properties were assigned as shown in Table 1. Cortical bone was defined with an elastic modulus of 15,000 MPa and a Poisson's ratio of 0.3. Grade IV titanium was characterized by an elastic modulus of 105,000 MPa and a Poisson's ratio of 0.33, while Ti6Al4V had an elastic modulus of 111,000 MPa and a Poisson's ratio of 0.34.

**Figure 3:**
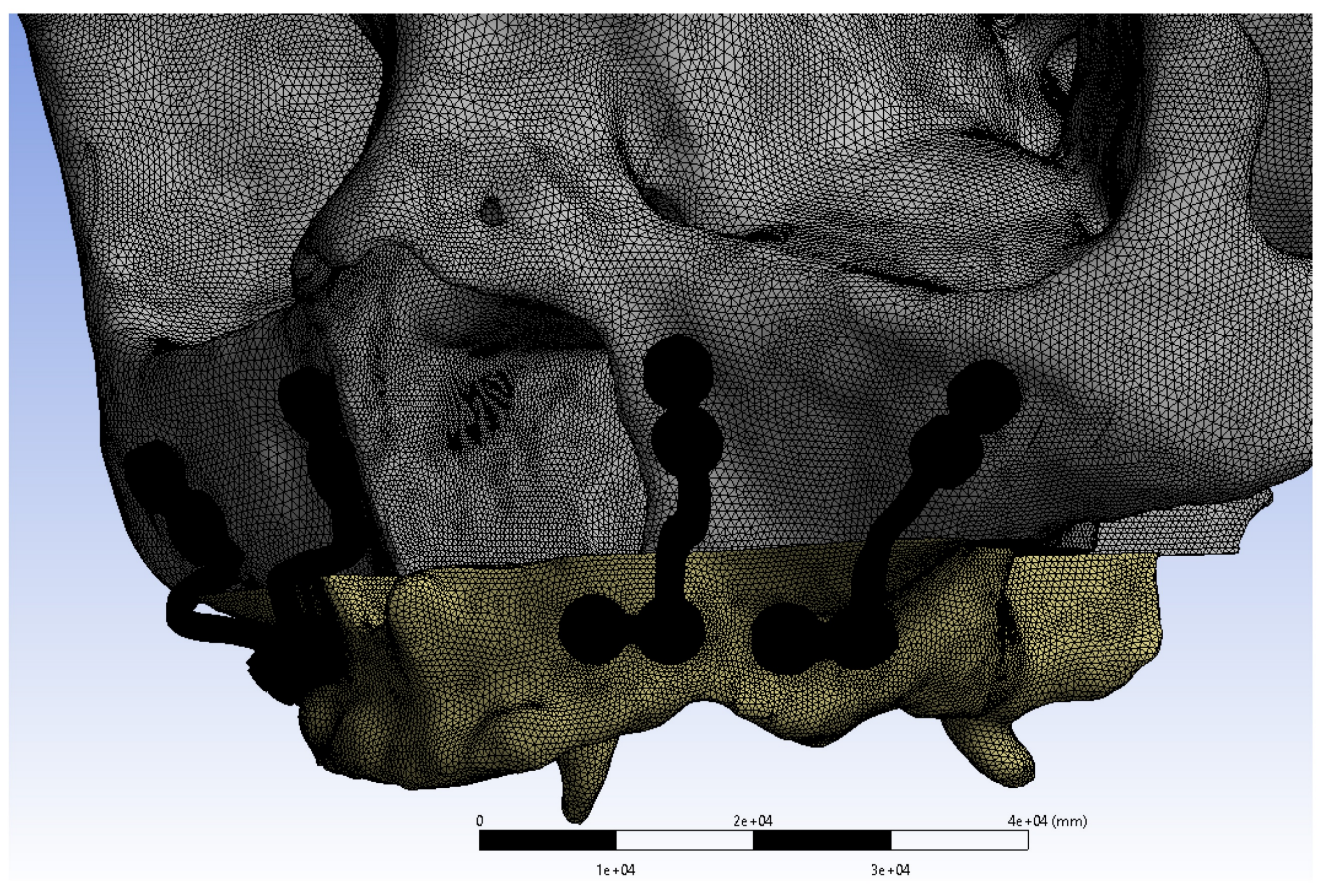
Mini plates mesh model image.

Mathematical models were generated by discretizing geometric models into small, simple elements known as meshes. Once the modelling process was completed in ANSYS SpaceClaim, the models were mathematically formulated in ANSYS Workbench and prepared for analysis. These models were then transferred to the LS-DYNA solver for computational simulations (Figure 3).

### Loading scenarios and boundary conditions

**Figure 4:**
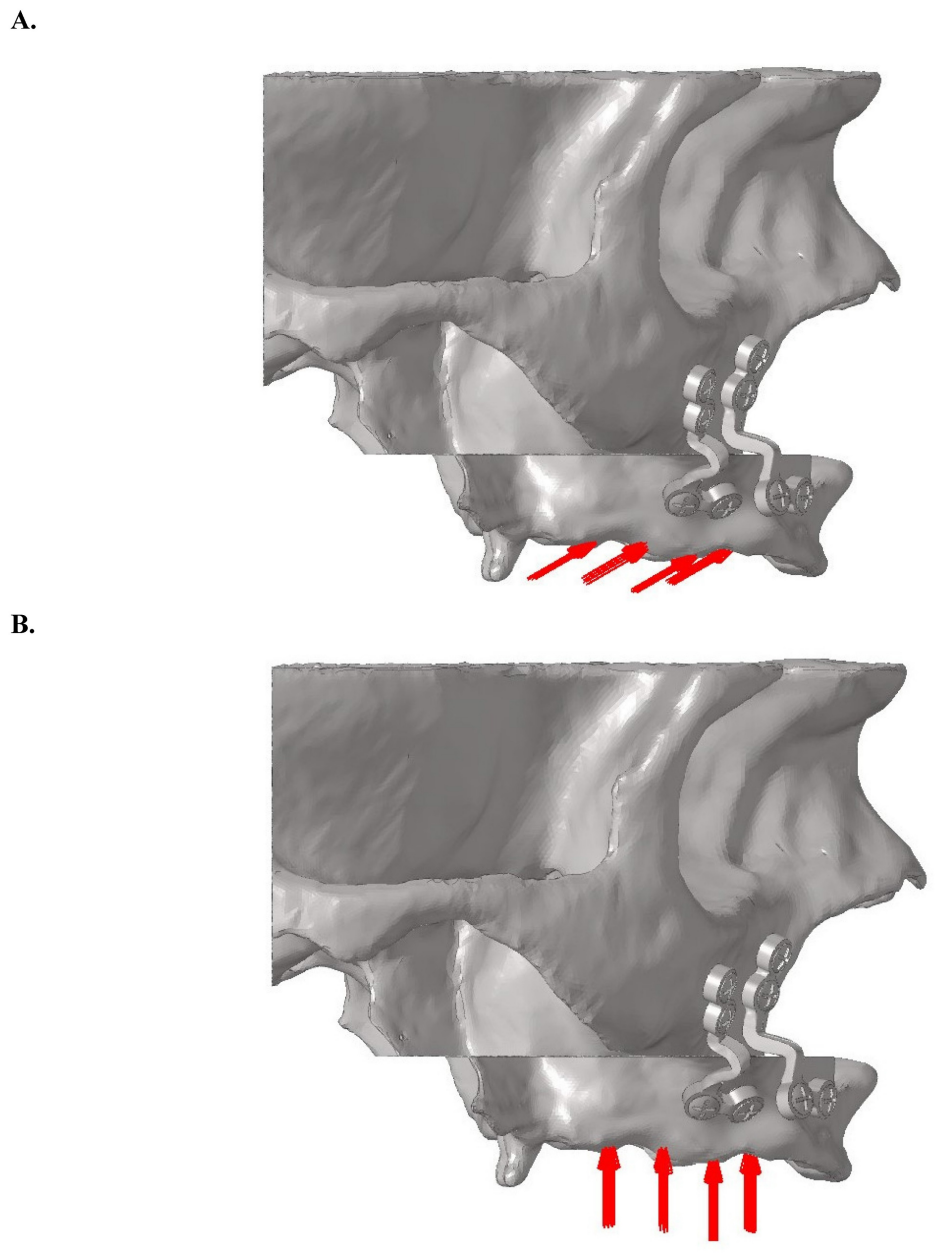

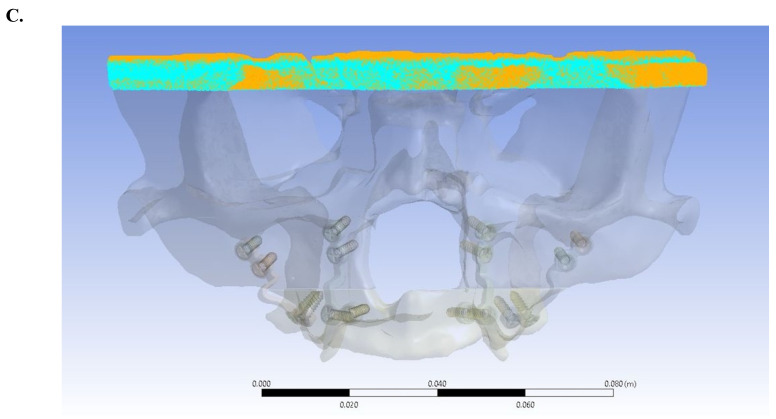
** A.** Oblique loading direction and force distribution on posterior teeth regions. **B.** Vertical loading direction and force distribution on posterior teeth regions.

For oblique loading, a bilateral force of 125 N was applied at a 30-degree angle from the palatal to buccal direction on the central fossae of the premolar and molar teeth. For vertical loading, a bilateral force of 125 N was applied perpendicular to the occlusal plane (Figure 4). The force magnitude of 125 N was selected based on physiological masticatory forces in the posterior maxilla, which typically range between 100 and 150 N in edentulous individuals 3,27. This value was consistent with previous FEA studies on orthognathic fixation 17,18, and represented a physiologically relevant functional load, particularly during the early postoperative period when occlusal forces are known to be reduced 14.

Separate vertical and oblique loading scenarios were simulated to independently assess their effects on stress distribution. Although simultaneous vector decomposition may more accurately represent complex mastication, distinct loading conditions provide a clearer biomechanical comparison without modifying the total applied force. This methodological approach was based on established modelling protocols in the literature 28,29.

The superior region of the maxilla was constrained to restrict movement along all axes. Nonlinear frictional contacts with a coefficient of 0.5 were defined between the screws and the plates/implants. Additionally, bonded contact conditions were applied between the screws and bone to simulate complete integration.

Nonlinear static analyses were conducted for both loading conditions in each fixation model, resulting in a total of four separate analyses. Displacement and stress distribution were assessed, with a particular focus on critical regions including the osteotomy site and screw interfaces.

## Results

**Figure 5:**
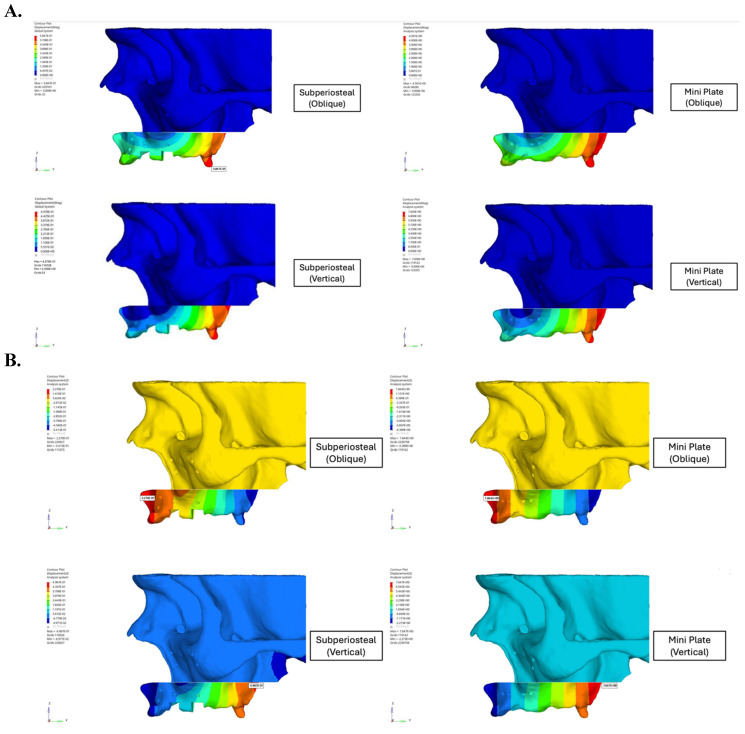
** A.** Displacement values ​​of custom subperiosteal implants and mini plates on the maxillary bone. **B.** Displacement values ​​of custom subperiosteal implants and mini plates on the maxillary bone.

This study demonstrated the significant biomechanical superiority of subperiosteal implants over traditional mini plates in maxillary orthognathic surgery. Displacement analysis revealed that the mini plate fixation group exhibited a maximum resultant displacement of 4.50 mm, nearly 10 times greater than the 0.58 mm observed in the subperiosteal implant group under identical loading conditions (Figure 5). This demonstrated enhanced stability provided by the customized implant design. Although oblique loading was emphasized due to its tendency to produce higher localized stress concentrations in the anterior regions, vertical loading resulted in greater overall displacement in both fixation models. The mini plate group exhibited a maximum displacement of 4.50 mm under vertical load, compared to 3.82 mm under oblique load. This suggested that vertical forces, which closely resemble actual compressive forces during mastication, pose a greater risk of structural deformation, particularly in traditional fixation systems. Conversely, the subperiosteal implant maintained low displacement values (0.58 mm under vertical load), indicating superior resistance and biomechanical stability under axial compressive stress.

**Figure 6:**
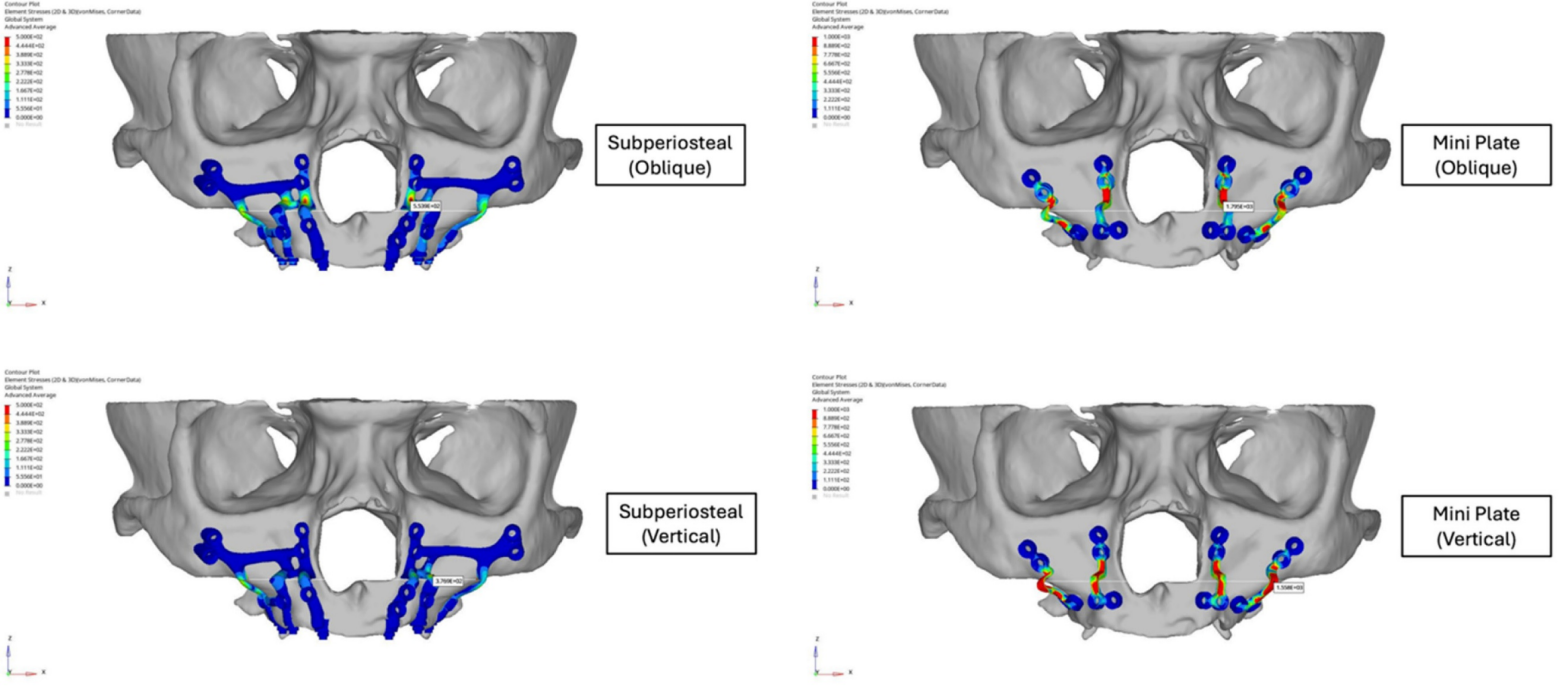
Stress distribution analysis of subperiosteal implants and mini plates.

Stress distribution analysis showed that critical stress levels on the mini plates were substantially higher compared to those on the subperiosteal implants. Under oblique loading, the maximum von Mises stress on the mini plates was approximately twice that of the subperiosteal implants. Similarly, under vertical loading, the stress observed in the mini plates was also approximately two times higher than in the subperiosteal implants (Figure 6). The stresses in the mini plates exceeded the yield strength of grade IV Titanium (480 MPa), particularly around the screws, where values reached up to 1,000 MPa. This indicated a risk of plastic deformation under functional loading, whereas the subperiosteal implants remained within the elastic range of Ti6Al4V (830-900 MPa), maintaining structural integrity under both loading conditions.

Mini plates, manufactured using traditional computer numerical control (CNC) methods with grade IV titanium, have been widely used in Le Fort surgeries for decades. In contrast, the relatively newer concept of custom orthognathic plates, produced through additive manufacturing methods utilizing Ti6Al4V, offered higher yield and ultimate tensile strength. The integration of subperiosteal implants with the design flexibility of custom orthognathic plates represents a novel approach in maxillary fixation.

**Figure 7:**
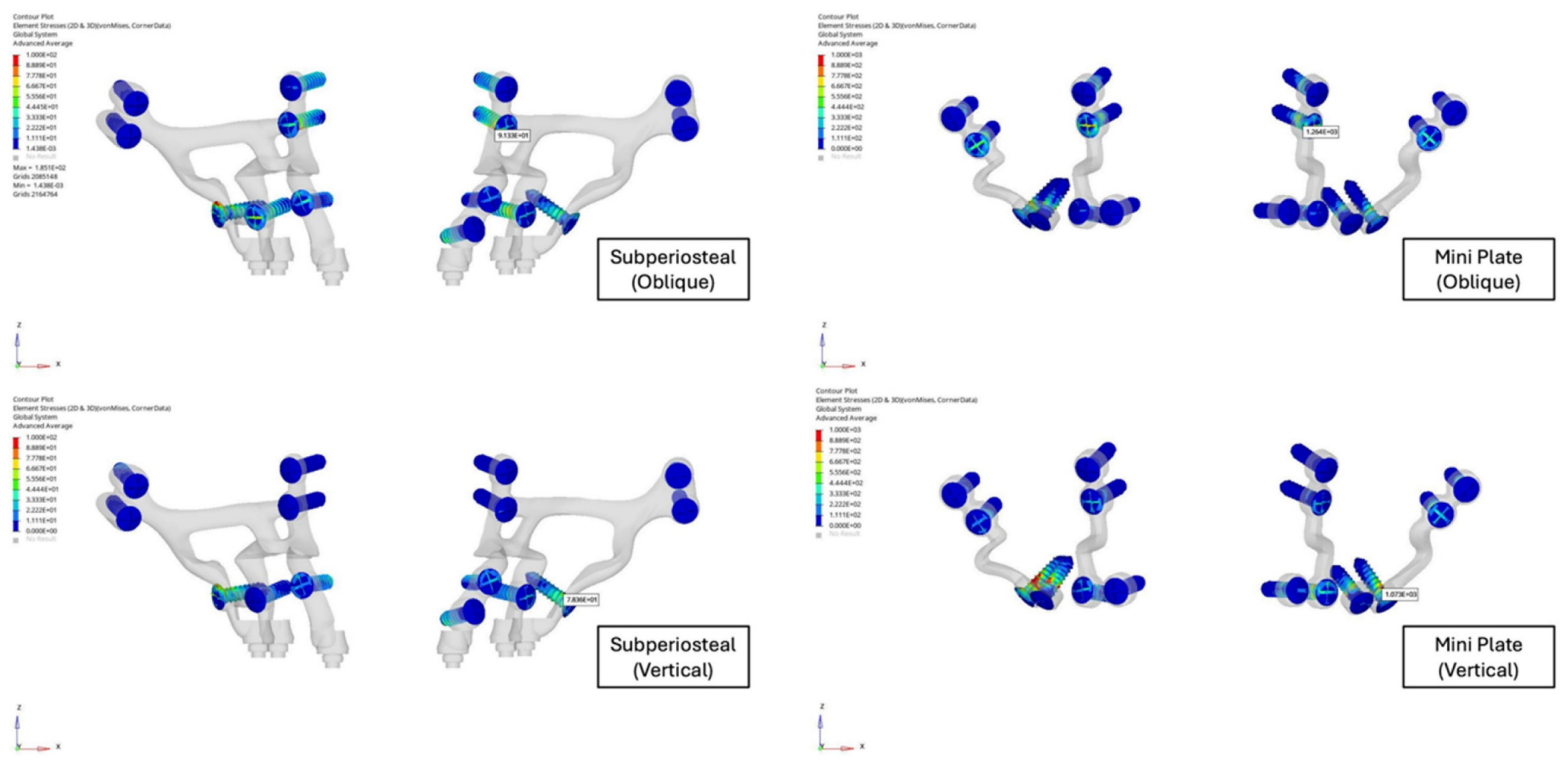
Stress levels of the screws of subperiosteal implants and mini plates.

The highest stress concentrations were observed near the anterior regions of the implants and plates during oblique loading and in the posterior regions during vertical loading. The screws in the subperiosteal implant group experienced significantly lower stress levels compared to those in the mini plate group, although in both cases screw stresses surpassed the yield strength of grade IV titanium (Figure 7). Bending forces and moments were identified as the primary contributors to stress concentration near the Le Fort osteotomy sites.

Overall, these findings highlighted the biomechanical advantages of subperiosteal implants, which exhibited superior stability, reduced stress concentrations, and enhanced resistance to deformation. These attributes suggested that subperiosteal implants present a promising alternative to traditional mini plates in orthognathic surgical applications.

## Discussion

As advancements in orthognathic surgery continue to redefine treatment paradigms, this study demonstrated the necessity of integrating biomechanical insights with cutting-edge technologies to enhance clinical outcomes. Orthognathic procedures, particularly in cases involving severe maxillary atrophy or edentulous patients, pose unique challenges that demand innovative solutions 30,31. This study demonstrated the biomechanical superiority of subperiosteal implants over traditional mini plates, aligning with previous studies that have reported the advantages of customized implant solutions for complex anatomical scenarios.

Subperiosteal implants, fabricated from Ti6Al4V, exhibited significantly greater yield and tensile strength compared to grade IV titanium mini plates. Stress distribution analysis revealed that mini plates consistently exceeded their material yield strength of 480 MPa under oblique and vertical loading conditions, predisposing them to plastic deformation. In contrast, subperiosteal implants remained within the elastic range of Ti6Al4V (830-900 MPa), demonstrating their ability to maintain structural integrity under comparable loading conditions. Furthermore, displacement analysis showed the biomechanical benefits of subperiosteal implants, demonstrating a tenfold reduction in displacement (0.58 mm) relative to mini plates (4.50 mm). This finding was consistent with the study of Alberts et al., which demonstrated that reduced displacement was critical for improving fixation longevity and mitigating the risk of implant failure 32. Additionally, the pronounced stress concentrations observed in the anterior and posterior regions of the osteotomy in mini plates were significantly alleviated by subperiosteal implants, ensuring a more uniform stress distribution. This observation corroborated the findings of Huang et al., who emphasized the importance of optimized implant design in reducing localized stresses and enhancing biomechanical performance 17.

Analysis of screw stresses revealed that in both fixation models, screws were subjected to stress levels exceeding the yield strength of grade IV titanium (480 MPa). However, the screws in the subperiosteal implant group exhibited significantly lower stress levels than those in the mini plate group. This distinction was important, as excessive stress on screws can lead to loosening, deformation, or failure over time, ultimately compromising the stability of the entire fixation system. Brunso et al. also demonstrated that reduced screw stress is indicative of optimized load distribution, which enhances the durability of fixation systems 13.

Stokbro et al. conducted an *in vitro* study comparing patient-specific 3D-printed plates with manually adapted commercial plates for Le Fort I osteotomy stabilization. Their findings indicated that 3D-printed plates exhibited superior mechanical performance, characterized by higher yield points and elastic modulus, enabling them to withstand greater forces compared to traditional plates 3. These findings were in agreement with the present study, where subperiosteal implants demonstrated similar biomechanical advantages due to their customized design and material properties. Collectively, these results highlighted the critical role of advanced manufacturing techniques in improving the stability and longevity of fixation systems in orthognathic surgery.

Ayhan and Özkeskin reported a case involving the rehabilitation of a severely atrophic maxilla in a patient with ectodermal dysplasia, utilizing Le Fort I advancement surgery in conjunction with subperiosteal implants 23. Their findings highlighted the effectiveness of subperiosteal implants in both fixation and prosthetic rehabilitation, demonstrating their capacity to provide long-term stability and functional restoration. This clinical case reinforced the real-world applicability of subperiosteal implants and validated their role in complex reconstructive scenarios.

Huang et al. conducted an FEA to evaluate the biomechanical behaviour of various mini plate fixations in maxillary advancement cases. Their study demonstrated that traditional mini plates are susceptible to plastic deformation under repeated bending, which negatively impacts stability. These findings aligned with the present study's observation that mini plates frequently exceeded their material yield strength under oblique and vertical loading, leading to deformation. Collectively, these results highlighted the limitations of traditional plates in maintaining long-term stability and reinforced the benefits of advanced implant materials and designs in optimizing biomechanical performance 17.

The distribution of screw stress was also closely associated with the implant's design and material properties. The quantitative analysis in this study revealed that subperiosteal implants reduced peak stress on screws by approximately 38% compared to mini plates under oblique loading and by 42% under vertical loading, demonstrating their superior ability to distribute loads more evenly. This more homogeneous stress distribution effectively minimized high-stress concentrations at the screw-plate interface, a common failure point in mini plates. Conversely, mini plates exhibited a tendency to concentrate stress in these critical regions, with stress values reaching up to 510 MPa, compared to 295 MPa in subperiosteal implants, thereby increasing the likelihood of mechanical failure 2,17,18. This observation was consistent with previous biomechanical models, which have demonstrated that excessive stress peaks at the screw-plate interface significantly compromise screw longevity under repetitive loading conditions. The reduction in stress concentration observed with subperiosteal implants supported their superior biomechanical integration, ultimately enhancing both stability and durability in clinical applications 19,28.

However, it is important to acknowledge that the observed differences in stress distribution between mini plates and subperiosteal implants may have been influenced not only by geometric design, but also by variations in material properties. While subperiosteal implants exhibited lower peak stress values, this advantage may be partly attributable to the superior mechanical strength of Ti6Al4V compared to grade IV titanium. Therefore, the presence of critical stress points in either system does not inherently indicate superior or inferior overall stress distribution. A more comprehensive evaluation should consider how stress propagates throughout the entire fixation system, accounting for both peak stress concentrations and the capacity to dissipate mechanical loads efficiently. Recognizing this as a limitation of the current study, we propose that future FEAs adopt a material-controlled approach in which both systems utilize the same alloy. This would facilitate a more precise assessment of the effects of implant geometry and design independent of material differences.

Despite the biomechanical limitations identified in this study, mini plates remain widely used in routine orthognathic surgery due to their long-standing clinical familiarity, intraoperative flexibility, and cost-effectiveness. In patients with sufficient bone volume and mild-to-moderate maxillary movements, they often provide adequate fixation within physiological limits. However, in edentulous patients, those with advanced alveolar atrophy, or cases involving large advancements, such as the 9 mm simulated in this study, the risk of elastic and plastic deformation increases significantly. Additionally, mini plates are typically contoured intraoperatively to conform to the underlying bone structure. Repeated bending of titanium during manual adaptation may introduce internal stresses and initiate microstructural fatigue, ultimately weakening the plate's mechanical performance over time 33. In contrast, patient-specific subperiosteal implants, which are fabricated preoperatively to match the patient's anatomical geometry, offer improved stress distribution and greater resistance to fatigue-related failures. Based on these findings, we suggest that subperiosteal implants may be better suited for high-load or complex cases where mini plates may be biomechanically insufficient. Traditional mini plate systems cost $150-$300 per set, whereas patient-specific subperiosteal implants range from $800 to $1,500 due to customization and additive manufacturing. Despite higher initial costs, their dual role in fixation and prosthetic support may reduce additional procedures, enhancing treatment efficiency and cost-effectiveness in complex cases.

Despite these promising results, certain limitations of this study must be acknowledged. Firstly, all screws and plates used in the models were standardized to 2 mm in diameter and 8 mm in length, which may not fully reflect the variability encountered in clinical practice. Secondly, the two fixation groups were fabricated from different materials, grade IV titanium for mini plates and Ti6Al4V for subperiosteal implants, each of which has distinct mechanical properties. These inherent material differences may have influenced the observed biomechanical performance, suggesting that future studies should aim to standardize material properties to more precisely isolate the effects of implant design and geometry. Additionally, while the finite element models used in this study provided comprehensive biomechanical insights, they represent simplified physiological conditions and may not fully capture the dynamic environment of the maxilla 34,35. To validate these findings, long-term clinical studies with larger patient cohorts are necessary to assess additional factors, such as osseointegration, soft tissue interactions, and patient-reported outcomes.

Although this study primarily focused on the biomechanical performance of fixation methods rather than clinical complications, it is important to clarify that the osteotomy technique itself does not inherently increase the risk of infection. When performed under standard sterile conditions with appropriate perioperative management, the likelihood of infection remains minimal and is more closely related to surgical hygiene and postoperative care than to the method of osteotomy. Previous studies have emphasized the importance of evaluating implant survival rates and complications in real-world clinical settings, particularly when introducing innovative implant designs 32,36,37.

Future research should explore hybrid implant designs that integrate the advantages of both traditional mini plates and subperiosteal implants, potentially combining structural stability with enhanced biological integration. Surface modifications, such as porous coatings or bioactive treatments, may further improve osseointegration and long-term implant performance 38,39. Additionally, comparing the cost-effectiveness and surgical efficiency of these novel solutions with existing fixation methods will be crucial in determining their broader clinical utility.

In conclusion, this study highlighted the superior biomechanical performance of subperiosteal implants, particularly in reducing displacement and mitigating stress concentrations compared to traditional mini plates. The analysis of screw performance further reinforced the critical role of optimized implant design in ensuring the longevity and stability of fixation systems. By minimizing screw stress and enhancing load distribution, subperiosteal implants present a transformative solution for complex maxillofacial reconstructions. However, despite these biomechanical advantages, traditional mini plates continue to be widely utilized in orthognathic surgery due to their long-standing clinical familiarity, ease of intraoperative application, and broad commercial availability. In cases with sufficient bone volume and moderate loading conditions, mini plates often provide adequate fixation within physiological limits. Their cost-effectiveness and established regulatory approval also contribute to their continued preference in many clinical settings.

Nonetheless, as additive manufacturing technologies become more accessible and patient-specific implant designs gain wider adoption, subperiosteal implants may emerge as a compelling alternative, particularly for anatomically and biomechanically challenging reconstructions. While mini plates remain relevant for routine applications, the findings of this study have highlighted the growing potential of subperiosteal implants to replace them in complex cases. These results emphasized the transformative impact of integrating advanced materials and additive manufacturing technologies in orthognathic surgery, paving the way for innovative, patient-specific solutions that can enhance clinical outcomes and expand the therapeutic possibilities in maxillofacial reconstruction.

## Figures and Tables

**Table 1:** Material properties of the analyzed models

Material	Elastic Modulus [MPa]	Poisson Ratio [V]	Elastic Limit [MPa]
Cortical Bone	15 000	0.3	120-150
Titanium (Grade 4)	105 000	0.33	480
Titanium (Ti6al4v)	111 000	0.34	830-900
